# Editorial: Crosstalk between bone and other cells

**DOI:** 10.3389/fphys.2023.1209053

**Published:** 2023-05-09

**Authors:** Narattaphol Charoenphandhu, Krittikan Chanpaisaeng, Jarinthorn Teerapornpuntakit, Kannikar Wongdee

**Affiliations:** ^1^ Center of Calcium and Bone Research (COCAB), Faculty of Science, Mahidol University, Bangkok, Thailand; ^2^ Department of Physiology, Faculty of Science, Mahidol University, Bangkok, Thailand; ^3^ Institute of Molecular Biosciences, Mahidol University, Nakhon Pathom, Thailand; ^4^ The Academy of Science, The Royal Society of Thailand, Bangkok, Thailand; ^5^ National Center for Genetic Engineering and Biotechnology (BIOTEC), National Science and Technology Development Agency, Pathum Thani, Thailand; ^6^ Department of Physiology, Faculty of Medical Science, Naresuan University, Phitsanulok, Thailand; ^7^ Faculty of Allied Health Sciences, Burapha University, Chonburi, Thailand

**Keywords:** bone, calcium, immune cells, maternal bone metabolism, myokine

Communications between osseous tissues and other organs are very common and essential for physiology of overall body system. For example, osteoblasts and osteocytes—now as endocrine cells (Figure 1)—are able to produce and release fibroblast growth factor (FGF)-23, which, in turn, regulates renal phosphate reabsorption (Vervloet, 2019; Agoro and White, 2023) and calcium transport across the intestinal epithelium (Khuituan et al., 2012; Rodrat et al., 2018; Wongdee et al., 2018; Wongdee et al., 2021). FGF-23 also downregulates the expression and activity of 25-hydroxyvitamin D 1α-hydroxylase (CYP27B1) in the renal proximal tubular cells, thereby reducing the action of 1,25-dihydroxyvitamin D_3_ (Perwad et al., 2007). During acute inflammation, osteocytes release certain mediators including C-terminal FGF-23 peptides to modulate hepatic hepcidin production and serum iron (Courbon et al., 2023). Moreover, osteocalcin or γ-carboxyglutamic acid-containing protein—as an osteoblast-derived endocrine factor—is capable of regulating pancreatic insulin production (Lee et al., 2007), adiponectin release from adipocytes (Lee et al., 2007) or testicular androgen biosynthesis (Karsenty and Oury, 2014).

**FIGURE 1:**
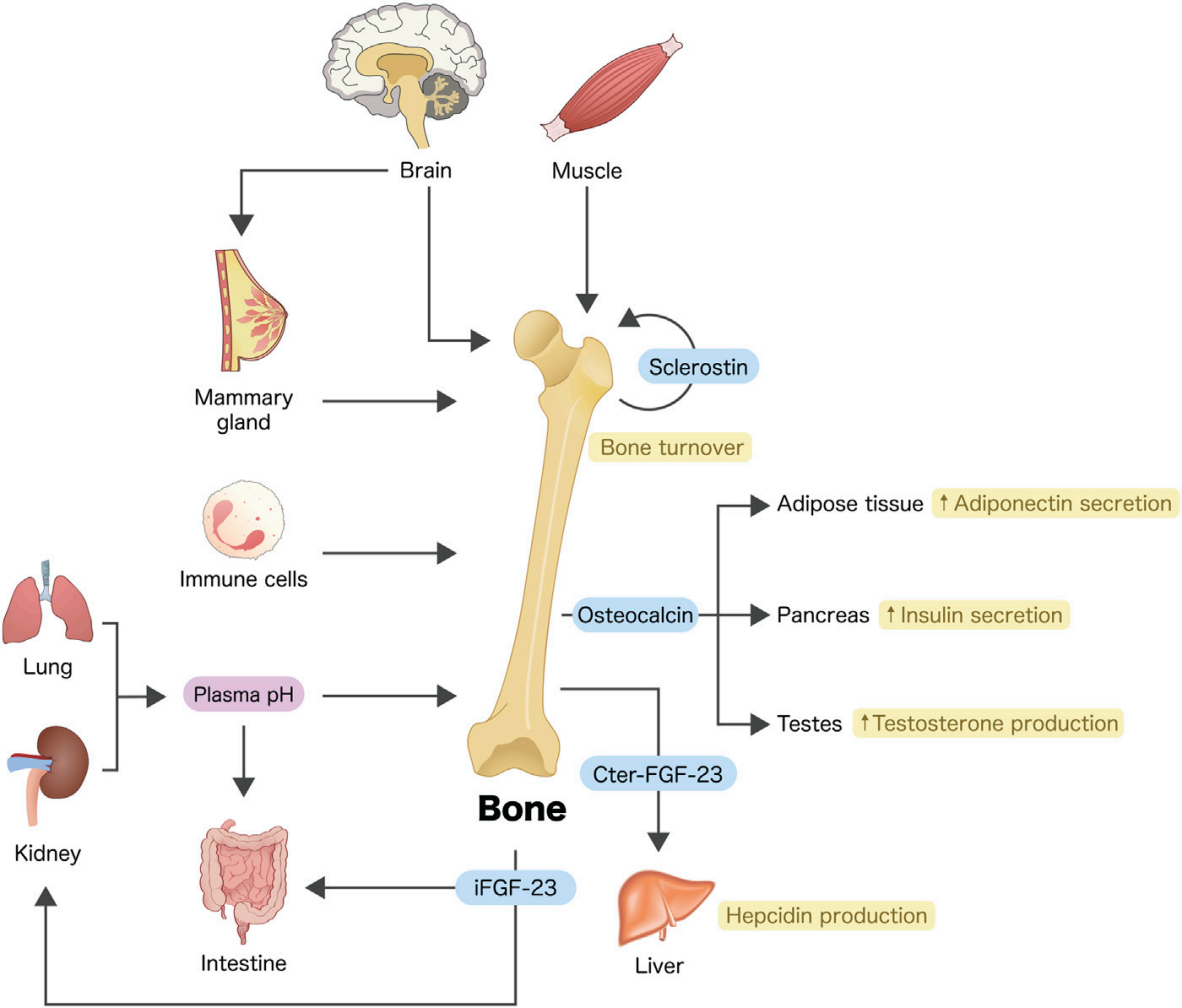
Crosstalks between bone and other organs or cells. Osteoblasts and/or osteocytes are considered endocrine cells that produce a number of bioactive molecules, e.g., osteocalcin, FGF-23 and sclerostin (see text for details). Some tissues or cells such as skeletal muscle, brain, immune cells, *etc.*, also produce bioactive molecules to modulate bone turnover. Arrows indicate communication, modulation and/or regulation. iFGF-23, intact fibroblast growth factor-23; Cter-FGF-23, C-terminal FGF-23 peptide.

On the other hand, several tissues, such as muscle and brain, also send signals to modulate bone remodeling (Rousseaud et al., 2016; Gomarasca et al., 2020). For instance, myokines (e.g., interleukin-6 and irisin) have been reported to positively regulate bone formation (Gomarasca et al., 2020), whereas central sympathetic outflow probably enhances bone resorption through β_2_-adrenergic receptor (Bonnet et al., 2008; Huang et al., 2009). Certain organs such as lung and kidney are principal regulators of plasma pH, a disturbance of which (e.g., metabolic acidosis) profoundly affects intestinal calcium absorption (Charoenphandhu et al., 2007) and osteoblast-mediated bone formation (Bushinsky and Krieger, 2022).

In the present Research Topic, there are publications that clearly point out the crosstalk between bone and some other tissues and cells, including the respiratory tissues, adipose tissue, mammary tissue, brain, and immune cells (e.g., monocytes, T-cells, *etc.*). Specifically, in an original article by Ivanova et al., the results corroborated that a disruption of Wnt-sclerostin pathway contributed to osteopathy (i.e., osteopenia and osteoporosis) in patients with Gaucher’s disease, a common lysosomal storage disease caused by acid β-glucocerebrosidase gene mutation with cellular accumulation of glucocerebroside. Another original article by Kurgan et al. demonstrated the effects of treadmill exercise on serum sclerostin levels in male mice. Sclerostin has been recognized as a secretory glycoprotein that strongly negates bone formation (Delgado-Calle et al., 2017; Liao et al., 2022), especially during mechanical unloading (Lin et al., 2009). It is predominantly expressed by osteocytes in order to suppress osteogenesis by regulating proliferation, differentiation, mineralization and apoptosis of osteoblasts (Winkler et al., 2003; van Bezooijen et al., 2004; Liao et al., 2022). Sclerostin is also expressed in some other tissues, e.g., cartilage, heart, kidney, and liver as well (Brunkow et al., 2001; Weivoda et al., 2017). Since Kurgan and others (Kurgan et al.) assessed serum sclerostin in exercising male mice and found a decrease in serum sclerostin in exercising group, mechanical loading during exercise is probably an efficient way to restrict sclerostin secretion from both osteocytes and adipose tissue (Kurgan et al.; Oniszczuk et al., 2022).

Generally, Wnt/β-catenin signaling plays a critical role in bone homeostasis by promoting osteoblastogenesis and bone formation. On the other hand, sclerostin—known as a Wnt signaling antagonist—binds to Wnt co-receptors, i.e., low-density lipoprotein receptor-related proteins 5 and 6 (LRP5 and LRP6), thereby preventing Wnt from binding to these co-receptor proteins. As a consequence, sclerostin blocks the Wnt downstream signaling pathway and downregulates the expression of genes involved in osteoblast commitment, such as *RUNX2* (Delgado-Calle et al., 2017). Several factors have been reported to suppress the expression of sclerostin including parathyroid hormone, estrogen as well as mechanical loading (Drake and Khosla, 2017). These factors thus act in concert with the Wnt/β-catenin signaling pathway to modulate bone homeostasis.

This Research Topic also contains review and mini-review articles that elaborate the crosstalk between bone and other tissues. Specifically, Ma and others (Ma et al.) explained how pulmonary tuberculosis, lung cancers, pollutant exposure [including particulate matter 2.5 (PM_2.5_)], asthma and chronic obstructive pulmonary disease are able to cause or aggravate osteoporosis. In a mini-review by He and Jiang, the interdependent interactions between immune cells (e.g., T cells, macrophages, NK cells, and dendritic cells) and cancer cells in bone microenvironment are well delineated. Lastly, Athonvarangkul and Wysolmerski discussed the physiological significances of the brain–breast–bone axis and maternal skeletal changes. A maternal pathological condition known as pregnancy/lactation-associated osteoporosis was also mentioned. Maternal central nervous system—particularly hypothalamus—controls bone metabolism by altering a number of humoral factors, e.g., prolactin, oxytocin and gonadotropin-releasing hormone, the latter of which is the salient regulator of circulating gonadotropins and estrogen. Prolactin not only enhances the intestinal calcium absorption (Charoenphandhu et al., 2009) but also modulates the expression of osteoblast-derived osteoclastogenic factors (Wongdee et al., 2011) and sclerostin production (Athonvarangkul and Wysolmerski).

Therefore, the present Research Topic is certainly able to shed some light on the crosstalks between bone and other distant cells, especially in lung and adipose tissue as well as cells in the brain–breast–bone axis (Figure 1). Indeed, interactions between bone cells and neighboring hematopoietic cells or stem cells within marrow tissue microenvironment are not uncommon and their detailed signaling networks are worth exploring.

## References

[B1] Agoro R. White K. E. (2023). Regulation of FGF23 production and phosphate metabolism by bone-kidney interactions. Nat. Rev. Nephrol. 19 (3), 185–193. 10.1038/s41581-022-00665-x 36624273

[B2] Bonnet N. Benhamou C. L. Malaval L. Goncalves C. Vico L. Eder V. (2008). Low dose beta-blocker prevents ovariectomy-induced bone loss in rats without affecting heart functions. J. Cell Physiol. 217 (3), 819–827. 10.1002/jcp.21564 18727092

[B3] Brunkow M. E. Gardner J. C. Van Ness J. Paeper B. W. Kovacevich B. R. Proll S. (2001). Bone dysplasia sclerosteosis results from loss of the *SOST* gene product, a novel cystine knot-containing protein. Am. J. Hum. Genet. 68 (3), 577–589. 10.1086/318811 11179006 PMC1274471

[B4] Bushinsky D. A. Krieger N. S. (2022). Effects of acid on bone. Kidney Int. 101 (6), 1160–1170. 10.1016/j.kint.2022.02.032 35351460 PMC9133222

[B5] Charoenphandhu N. Nakkrasae L. I. Kraidith K. Teerapornpuntakit J. Thongchote K. Thongon N. (2009). Two-step stimulation of intestinal Ca^2+^ absorption during lactation by long-term prolactin exposure and suckling-induced prolactin surge. Am. J. Physiol. Endocrinol. Metab. 297 (3), E609–E619. 10.1152/ajpendo.00347.2009 19567804

[B6] Charoenphandhu N. Wongdee K. Tudpor K. Pandaranandaka J. Krishnamra N. (2007). Chronic metabolic acidosis upregulated claudin mRNA expression in the duodenal enterocytes of female rats. Life Sci. 80 (19), 1729–1737. 10.1016/j.lfs.2007.01.063 17383680

[B7] Courbon G. Thomas J. J. Martinez Calle M. Wang X. Spindler J. Von Drasek J. (2023). Bone-derived C-terminal FGF23 cleaved peptides increase iron availability in acute inflammation. Blood 2022, 18475. 10.1182/blood.2022018475 PMC10356820 37053547

[B8] Delgado-Calle J. Sato A. Y. Bellido T. (2017). Role and mechanism of action of sclerostin in bone. Bone 96, 29–37. 10.1016/j.bone.2016.10.007 27742498 PMC5328835

[B9] Drake M. T. Khosla S. (2017). Hormonal and systemic regulation of sclerostin. Bone 96, 8–17. 10.1016/j.bone.2016.12.004 27965160 PMC5329134

[B10] Gomarasca M. Banfi G. Lombardi G. (2020). Myokines: The endocrine coupling of skeletal muscle and bone. Adv. Clin. Chem. 94, 155–218. 10.1016/bs.acc.2019.07.010 31952571

[B11] Huang H. H. Brennan T. C. Muir M. M. Mason R. S. (2009). Functional α_1_-and β_2_-adrenergic receptors in human osteoblasts. J. Cell Physiol. 220 (1), 267–275. 10.1002/jcp.21761 19334040

[B12] Karsenty G. Oury F. (2014). Regulation of male fertility by the bone-derived hormone osteocalcin. Mol. Cell Endocrinol. 382 (1), 521–526. 10.1016/j.mce.2013.10.008 24145129 PMC3850748

[B13] Khuituan P. Teerapornpuntakit J. Wongdee K. Suntornsaratoon P. Konthapakdee N. Sangsaksri J. (2012). Fibroblast growth factor-23 abolishes 1,25-dihydroxyvitamin D_3_-enhanced duodenal calcium transport in male mice. Am. J. Physiol. Endocrinol. Metab. 302 (8), E903–E913. 10.1152/ajpendo.00620.2011 22275752

[B14] Lee N. K. Sowa H. Hinoi E. Ferron M. Ahn J. D. Confavreux C. (2007). Endocrine regulation of energy metabolism by the skeleton. Cell 130 (3), 456–469. 10.1016/j.cell.2007.05.047 17693256 PMC2013746

[B15] Liao C. Liang S. Wang Y. Zhong T. Liu X. (2022). Sclerostin is a promising therapeutic target for oral inflammation and regenerative dentistry. J. Transl. Med. 20 (1), 221. 10.1186/s12967-022-03417-4 35562828 PMC9102262

[B16] Lin C. Jiang X. Dai Z. Guo X. Weng T. Wang J. (2009). Sclerostin mediates bone response to mechanical unloading through antagonizing Wnt/beta-catenin signaling. J. Bone Min. Res. 24 (10), 1651–1661. 10.1359/jbmr.090411 19419300

[B17] Oniszczuk A. Kaczmarek A. Kaczmarek M. Ciałowicz M. Arslan E. Silva A. F. (2022). Sclerostin as a biomarker of physical exercise in osteoporosis: A narrative review. Front. Endocrinol. (Lausanne). 13, 954895. 10.3389/fendo.2022.954895 36545331 PMC9760825

[B18] Perwad F. Zhang M. Y. Tenenhouse H. S. Portale A. A. (2007). Fibroblast growth factor 23 impairs phosphorus and vitamin D metabolism *in vivo* and suppresses 25-hydroxyvitamin D-1alpha-hydroxylase expression *in vitro* . Am. J. Physiol. Ren. Physiol. 293 (5), F1577–F1583. 10.1152/ajprenal.00463.2006 17699549

[B19] Rodrat M. Wongdee K. Panupinthu N. Thongbunchoo J. Teerapornpuntakit J. Krishnamra N. (2018). Prolonged exposure to 1,25(OH)_2_D_3_ and high ionized calcium induces FGF-23 production in intestinal epithelium-like caco-2 monolayer: A local negative feedback for preventing excessive calcium transport. Arch. Biochem. Biophys. 640, 10–16. 10.1016/j.abb.2017.12.022 29317227

[B20] Rousseaud A. Moriceau S. Ramos-Brossier M. Oury F. (2016). Bone-brain crosstalk and potential associated diseases. Horm. Mol. Biol. Clin. Investig. 28 (2), 69–83. 10.1515/hmbci-2016-0030 27626767

[B21] van Bezooijen R. L. Roelen B. A. Visser A. van der Wee-Pals L. de Wilt E. Karperien M. (2004). Sclerostin is an osteocyte-expressed negative regulator of bone formation, but not a classical BMP antagonist. J. Exp. Med. 199 (6), 805–814. 10.1084/jem.20031454 15024046 PMC2212719

[B22] Vervloet M. (2019). Renal and extrarenal effects of fibroblast growth factor 23. Nat. Rev. Nephrol. 15 (2), 109–120. 10.1038/s41581-018-0087-2 30514976

[B23] Weivoda M. M. Youssef S. J. Oursler M. J. (2017). Sclerostin expression and functions beyond the osteocyte. Bone 96, 45–50. 10.1016/j.bone.2016.11.024 27888056 PMC5328839

[B24] Winkler D. G. Sutherland M. K. Geoghegan J. C. Yu C. Hayes T. Skonier J. E. (2003). Osteocyte control of bone formation via sclerostin, a novel BMP antagonist. EMBO J. 22 (23), 6267–6276. 10.1093/emboj/cdg599 14633986 PMC291840

[B25] Wongdee K. Chanpaisaeng K. Teerapornpuntakit J. Charoenphandhu N. (2021). Intestinal calcium absorption. Compr. Physiol. 11 (3), 2047–2073. 10.1002/cphy.c200014 34058017

[B26] Wongdee K. Rodrat M. Keadsai C. Jantarajit W. Teerapornpuntakit J. Thongbunchoo J. (2018). Activation of calcium-sensing receptor by allosteric agonists cinacalcet and AC-265347 abolishes the 1,25(OH)_2_)D_3_-induced Ca^2+^ transport: Evidence that explains how the intestine prevents excessive Ca^2+^ absorption. Arch. Biochem. Biophys. 657, 15–22. 10.1016/j.abb.2018.09.004 30217510

[B27] Wongdee K. Tulalamba W. Thongbunchoo J. Krishnamra N. Charoenphandhu N. (2011). Prolactin alters the mRNA expression of osteoblast-derived osteoclastogenic factors in osteoblast-like UMR106 cells. Mol. Cell Biochem. 349 (1–2), 195–204. 10.1007/s11010-010-0674-4 21116687

